# Metastatic and non-metastatic melanoma imaging using Sgc8-c aptamer PTK7-recognizer

**DOI:** 10.1038/s41598-021-98828-6

**Published:** 2021-10-07

**Authors:** Estefanía Sicco, Amy Mónaco, Marcelo Fernandez, María Moreno, Victoria Calzada, Hugo Cerecetto

**Affiliations:** 1 Área de Radiofarmacia, Centro de Investigaciones Nucleares, Facultad de Ciencias, Universidad de La República, 11400 Montevideo, Uruguay; 2 Departamento de Desarrollo Biotecnológico, Instituto de Higiene, Facultad de Medicina, Universidad de La República, 11600 Montevideo, Uruguay; 3 Laboratorio de Experimentación Animal, Centro de Investigaciones Nucleares, Facultad de Ciencias, Universidad de La Republica, 11400 Montevideo, Uruguay

**Keywords:** Cancer imaging, Biomarkers, Molecular medicine, Oncology

## Abstract

Melanoma is one of the most aggressive and deadly skin cancers, and although histopathological criteria are used for its prognosis, biomarkers are necessary to identify the different evolution stages. The applications of molecular imaging include the in vivo diagnosis of cancer with probes that recognize the tumor-biomarkers specific expression allowing external image acquisitions and evaluation of the biological process in quali-quantitative ways. Aptamers are oligonucleotides that recognize targets with high affinity and specificity presenting advantages that make them interesting molecular imaging probes. Sgc8-c (DNA-aptamer) selectively recognizes PTK7-receptor overexpressed in various types of tumors. Herein, Sgc8-c was evaluated, for the first time, in a metastatic melanoma model as molecular imaging probe for in vivo diagnostic, as well as in a non-metastatic melanoma model. Firstly, two probes, radio- and fluorescent-probe, were in vitro evaluated verifying the high specific PTK7 recognition and its internalization in tumor cells by the endosomal route. Secondly, in vivo proof of concept was performed in animal tumor models. In addition, they have rapid clearance from blood exhibiting excellent target (tumor)/non-target organ ratios. Furthermore, optimal biodistribution was observed 24 h after probes injections accumulating almost exclusively in the tumor tissue. Sgc8-c is a potential tool for their specific use in the early detection of melanoma.

## Introduction

Melanoma is one of the most aggressive and deadly types of skin cancer^1,2^, with an annual increase in incidence during the last decade between 15 and 25 per 100,000 individuals^3^. Although histopathological criteria such as tumor thickness, mitotic rate, histologic subtype and ulceration^4,5^, are usually used for its prognosis, biomarkers are necessary to identify whether the primary melanoma has metastasized or even differentiate the stages of its evolution^6,7^. Malignant melanomas have been reported to have increased activity of protein tyrosine kinase 7 (PTK7)^8,9^. This membrane receptor is highly conserved in different species and is involved in signal transduction pathways that mediate cell growth, cell polarity, differentiation, and survival^10,11^. However, PTK7 may participate as a co-receptor and its protection by type 1 membrane metalloprotease is implicated in the progression of cancer^12,13^. PTK7 has also been shown to be a key regulator in the Wnt/β-Catenin or Wnt/planar cell polarity pathway, and correlates with aggressive clinicopathological characteristics in cancer^14,15^. In addition, this receptor is overexpressed in different types of leukemia, colon, lung, prostate, breast,Gastric tumors, and even metastases^16–22^. Furthermore, it participates in the migration and endothelial invasion of tumor cells^23–25^.

It is possible to identify the expression of tumor biomarkers using molecular imaging, a non-invasive technique that manages to evaluate the strategies for in vivo administration of the tumor target^26,27^. Molecular imaging consists of in vivo visualization, characterization and measurement of biological processes at the cellular or molecular level^27,28^, and is a very useful tool for diagnosing cancer. The use of probes that have optimal imaging characteristics provides clinically essential information for this disease, which would allow a correct selection of the treatment to be followed and the monitoring of its effects^29^.

In addition, aptamers have been used as a component of molecular imaging probes since they have the ability to bind, through non-covalent interaction bonds, with high affinity and specificity for a molecular target^30,31^. They are oligonucleotides (ssDNAs or RNAs) that have a three-dimensional structure characterized by loops, stems or hairpins. Aptamers have properties equivalent to antibodies in terms of their recognition by the molecular target, however they offer great advantages. The chemical synthetic process of aptamers allows a low cost production and no batch to batch variability^32,33^. They have physicochemical properties, such as stability at different temperatures and pH^34,35^, and can be easily modified to continue improving their biological stability and pharmacokinetics^26,36,37^.Furthermore, aptamers molecular weight (~ 15000 Da) and charge provide rapid penetration into target tissues and elimination from the body^31,38^. Also, aptamers do not generate immunogenicity or toxic effects. Therefore, the characteristics of aptamers provide great advantages for their use in the development of new molecular imaging agents^39^.

Previously, we have modified the Sgc8-c aptamer to generate molecular imaging probes in the diagnosis of cancer^39–42^. The Sgc8-c aptamer is DNA (41 nt) and selectively recognizes the PKT7 receptor with a *K*_d_ = 0.78 nM^10,43^. We have developed potential molecular imaging probes in different tumor models, through in vitro and in vivo evaluation, with an emphasis on hematological diseases. In this sense, radiolabelled probes (Sgc8-c-NOTA-^67^Ga, Sgc8-c-DOTA-^67^Ga, Sgc8-c-HYNIC-^99m^Tc) have been reported to have better tissue penetration and ability to accurately measure tissue, and as a result, it allows quantitative images of the whole body^39–42^. Likewise, the fluorescent probe (Sgc8-c-Alexa647) allows the generation of optical images in the near infrared region with little interference, achieving optimal contrasts due to the molecules present in the tissues, that do not exhibit high absorption in that spectral region^39,40^. Different versatile platforms have been reported, where the conjugation of Sgc8-c aptamer enhances the cellular uptake capability of the material, achieving specific images in vivo^44–46^, even in deep tissues^45^. These features are very useful to perform, for example, guided surgeries in real time^47,48^. Using the appropriate imaging probes, it is even possible to identify metastases^49^. Recently, we have studied these probes as imaging tools in a melanoma model, obtaining interesting results regarding tumor uptake^39^. Moreover, the sensitive and effective detection of PTK7 may represent a good strategy in the early diagnosis of melanoma. Based on this, the present work evaluates for the first time two potential different probes developed with of Sgc8-c in the metastatic melanoma model. Likewise, it also delves into another non-metastatic melanoma model, in order to optimize biological control methods, both biodistribution and imaging, achieving a sensitive and effective detection of PTK7 with these probes.

## Results and discussion

### PTK7 expression in B16F10 cells

**Figure 1:**
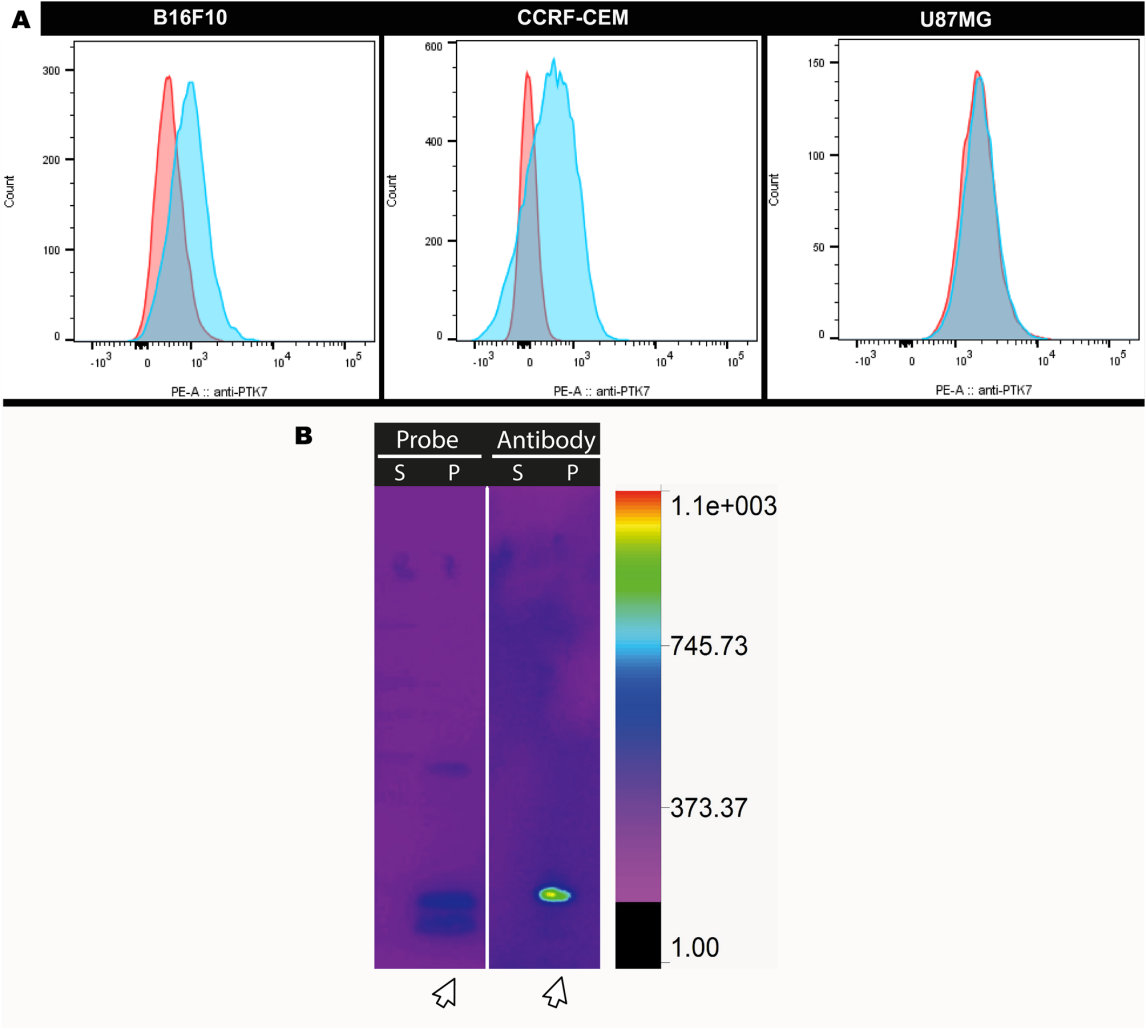
(**A**) Flow cytometry with cell lines B16F10, CCRF-CEM and U87MG. Histograms of cell lines after for incubation with anti-PTK7-PE antibody (in blue) and control (in red). (**B**) Grouping of Western blot cropped of proteins extracted from the supernatant (S) and pellet (P) of B16F10 cells culture. Incubation was performed with the Sgc8-c-Alexa647 probe (Probe) and with the anti-PTK7-PE antibody (Antibody). Arrow indicates the presence of PTK7 only in the cell pellet. White dividing line indicates different gels (performed in parallel) and exposures (full-length gels are shown in Fig. S1 in Supporting Information).

Firstly, we evaluated the presence of PTK7 in metastatic melanoma B16F10 cells by flow cytometry and Western blot, as it was previously evaluated in non-metastatic B16F1 melanoma cells^39,40^. Flow cytometry assays performed with a commercial anti-PTK7 antibody as probe revealed that approximately 40% of the B16F10 cells expressed detectable levels of PTK7. Of note, near to 80% of the positive control CCRF-CEM cells stained for PTK7, while the negative control, U87MG cells, showed null signal (Fig. 1A). Additionally, the Western blot studies confirmed the presence of PTK7 receptor on B16F10 cells (Fig. 1B, full-length gels are shown in Fig. S1 in Supporting Information). In this assay, it was observed that the probe also recognizes two fractions of PTK7. It has been reported in different cell lines that this receptor has different cleavage sites, which generate different matching cell-associated forms^13,50^.

### In vitro binding studies

As it was previously described^39,40^, Sgc8-c-NOTA-^67^Ga and Sgc8-c-Alexa647 probes were stable, according to RP-HPLC and gel electrophoresis results, in water up to 75 °C for 30 min of incubation and in addition the integrity of these conjugates under different storage conditions was confirmed. The observed electrophoresis profiles were consistent with the absence of low molecular weight fragmentation. Furthermore, for the precursor Sgc8-c-NOTA, the probe Sgc8-c-Alexa647 and as well as the original aptamer Sgc8-c-NH_2_, functional stabilities in DNase I, after 15 min and 2 h of incubation was studied observing similar level of degradation. After 15 min of incubation with the enzyme, it was found that Sgc8-c-NOTA was more resistant to degradation than the fluorescent probe (Sgc8-c-Alexa647). After 2 h, for all the samples, there was still aptamer without degradation. The behavior on fetal bovine serum (FBS) was also studied resulted in > 90% serum protein binding, and 66% instability^40^.

Two different strategies were employed to analyze the ability of Sgc8-c probes to interact to B16F10: the use of the radiolabelled probe (Sgc8-c-NOTA-^67^Ga) that can be measured byGamma counter system, and the use of the fluorescent-probe (Sgc8-c-Alexa647), which allows analysis by flow cytometry and Western blot.

**Figure 2:**
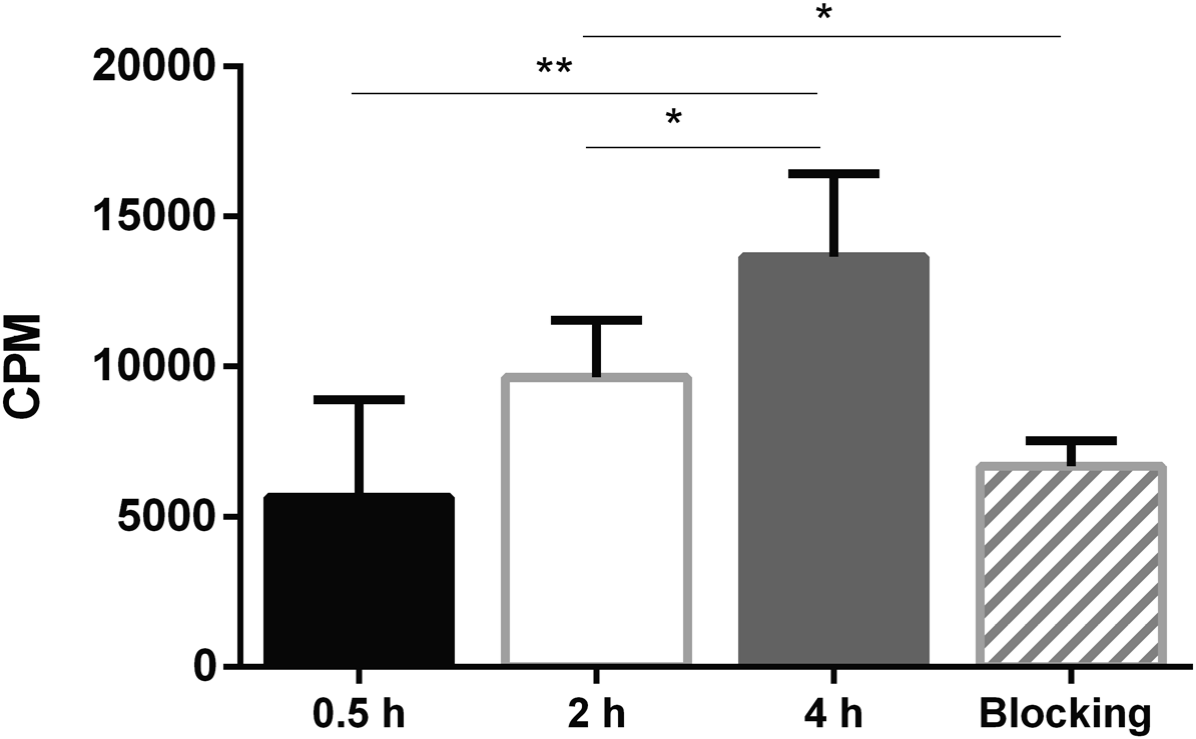
Binding assay with Sgc8-c-NOTA-^67^Ga in the B16F10 cell line. The probe was incubated for 0.5, 2 and 4 h. Blocking: represents the competition test performed by incubating the cells with an excess of unlabeled aptamer (Sgc8-c-NH_2_). **p < 0.01, *p < 0.05 (Student's *t* test).

The results showed that the Sgc8-c-NOTA-^67^Ga probe binds to the B16F10 cell line. It was found that the binding percentage increased with time with significant differences at 4 h of incubation (Fig. 2). Regarding the blocking test of cells with the unlabeled aptamer, it was observed that the percentage of binding to the labeled probe decreases (compare 2 h of incubation and blocking incubation, *p* < 0.05, Fig. 2) indicating that there was a competition between both compounds confirming the probe interaction with PTK7.

**Figure 3:**
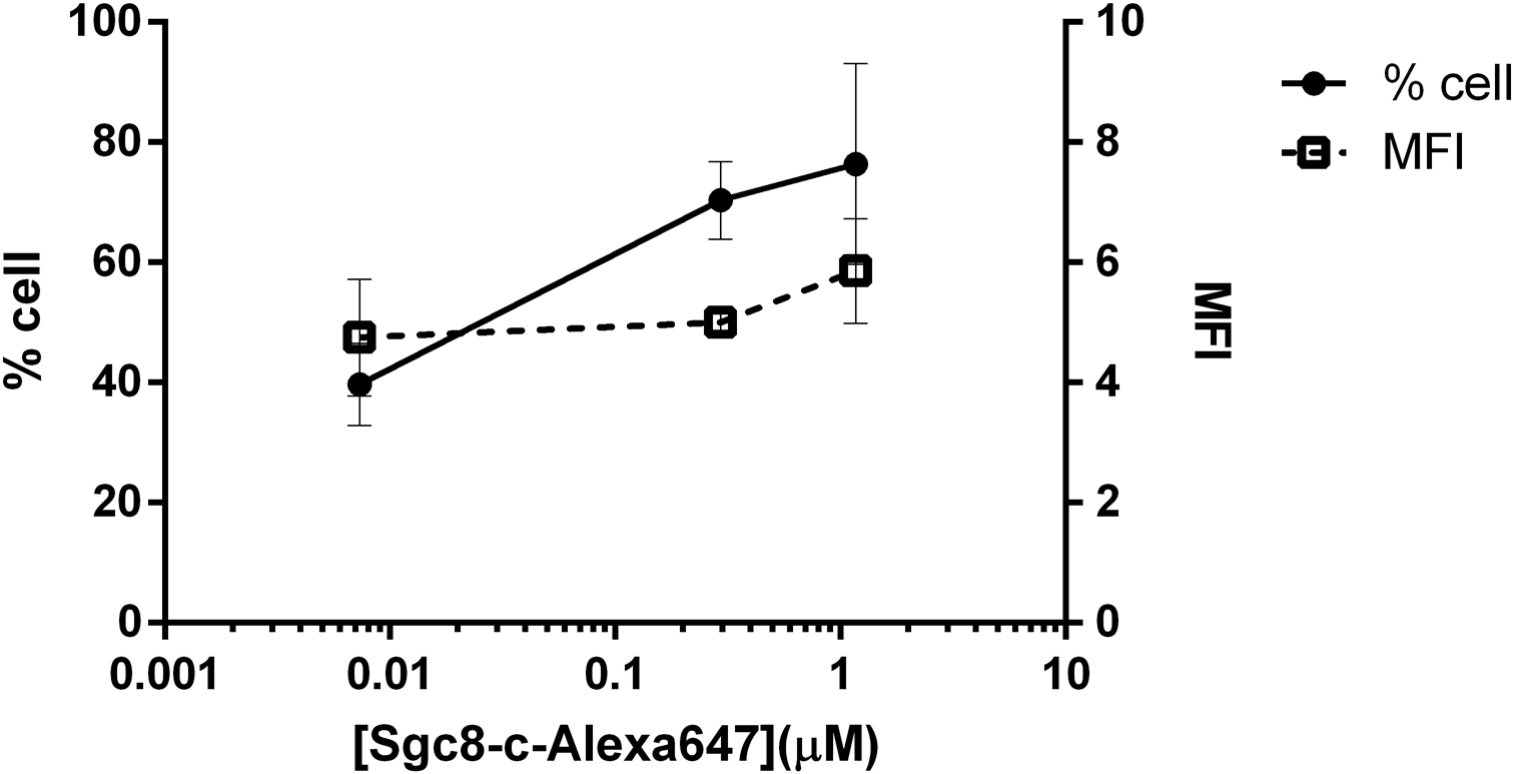
Flow cytometry of B16F10 cell line treated with Sgc8-c-Alexa647. Percentage of positive cells and specific MFI after incubation with the fluorescent probe.

For fluorescent flow cytometry assays, melanoma B16F10 cells were incubated with different concentrations of Sgc8-c-Alexa647. Figure 3 shows the percentage of positive cells and the specific mean fluorescence index (MFI). Results showed that the percentage of PTK7 positive cells is dependent on probe concentration, reaching a maximum of 80% of B16F10 cells with approximately 0.5 μM. Furthermore, the MFI indicates the amount of the probe that binds to PTK7, more precisely, the abundance of proteins at individual population cell level^51^, thus this suggests that the B16F10 cell line expresses significant high levels of the PTK7 receptor (Fig. 3).

According to cytometry analysis, saturation concentration was reached at 0.3 μM of aptamer without achieving 100% of the B16F10 cells. This phenomenon could be explained by different cell division and differentiation stages in cell culture. In addition, as mentioned above, a molecular cleavage phenomenon has been described upon Sgc8-PTK7 interaction^46^. If the cleaved PTK7 receptor had been excreted into the supernatant of the medium, as result of cleavage mechanism of the aptamer-PTK7 complex, it would be the cause of not reaching saturation when the process was analyzed by flow cytometry. Then, if the cleaved PTK7 receptor is excreted into the supernatant of the medium, its detection by flow cytometry would not be possible. However, all experiments were performed in ice so the chances of cleavage are almost null. Besides, Western blot studies using supernatants rejected this hypothesis. The results indicated that both the antibody and the fluorescent probe only recognize proteins from the cell pellet (Fig. 1B) showing that there was no detectable molecular cleavage in the supernatant. Based on these results, we propose the hypothesis that the rapid complex internalization does not allowing saturation due to the absence of the membrane receptor^53^. For this reason, fluorescent confocal microscopy studies were performed to demonstrate the internalization of the receptor (see below).

### In vivo binding studies

In order to perform the in vivo biological studies we first analyzed the ability of the Sgc-8-c-Alexa647 probe to recognize in vivo the PTK7 presence in target organs. For this, flow cytometry studies were performed on B16F10-tumors, and liver, spleen and bone marrows as negative controls. The results showed that the fluorescent-probe marked, from early times, tumor cells al high levels, while in the rest of the non-target organs the percentage of positive cells was very low (Table 1), showing that the probe binds specifically to the PTK7 receptors that are on melanoma cells. In these studies, it is interesting to observe that the union of the probe to the tumor cells is maintained for a longer time than in the other non-target organs, without observing a significant tendency. This could be due to tissue homogenization to analyze the cells by flow cytometry, because everything that is retained in the extracellular matrix or contained in the different tissues is lost, while observing only the specific binding of the probes in the single cells. Additional information can be observed with in vivo imaging and biodistribution studies.

**Table 1:** Percentage of positive cells for in vivo exposed organs to probe Sgc8-c-Alexa647 analyzed by flow cytometry.

Organ/time (h)	Percentage of positive cells (%)		
	0.5	2	24
Liver	3.80 ± 2.51	0.70 ± 0.35	0.30 ± 0.17
Spleen	1.10 ± 0.40	2.20 ± 1.15	0.60 ± 0.15
Bone marrow	0.93 ± 0.25	1.40 ± 0.49	0.60 ± 0.21
B16F10-tumor	86.40 ± 3.67	81.20 ± 4.55	82.80 ± 5.84

### Confocal microscopy

**Figure 4:**
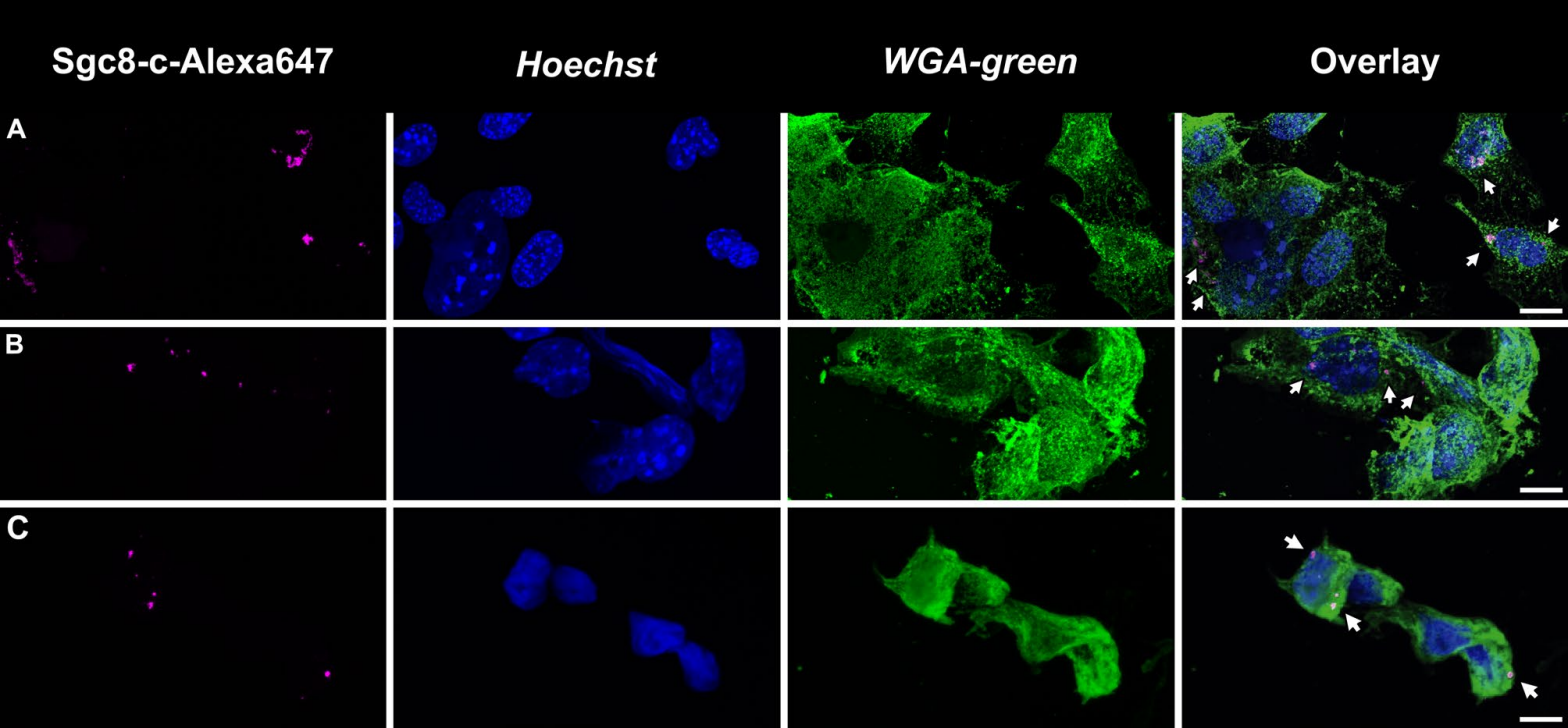
Confocal microscopy of B16F10 tumor cells incubated with fluorescent probe Sgc8-c-Alexa647. Cells were incubated with the probe for (**A**) 2 h, (**B**) 4 h, and (**C**) 16 h. Magenta: Sgc8-c-Alexa647 (white arrows), Blue: *Hoechst* and Green: *WGA-green*. Scale bar: 10 µm.

Another mechanism proposed for Sgc8-PTK7 interaction is complex internalization^47^. For this reason, fluorescent confocal microscopy studies were performed to demonstrate the internalization of the receptor on the studied tumor cells. To perform confocal microscopy assays, B16F10 tumor cells were incubated with the Sgc8-c-Alexa647 probe during different times. At early time point, it was evident that the Sgc8-c-Alexa647 probe was internalized by the cells (Fig. 4).

**Figure 5:**
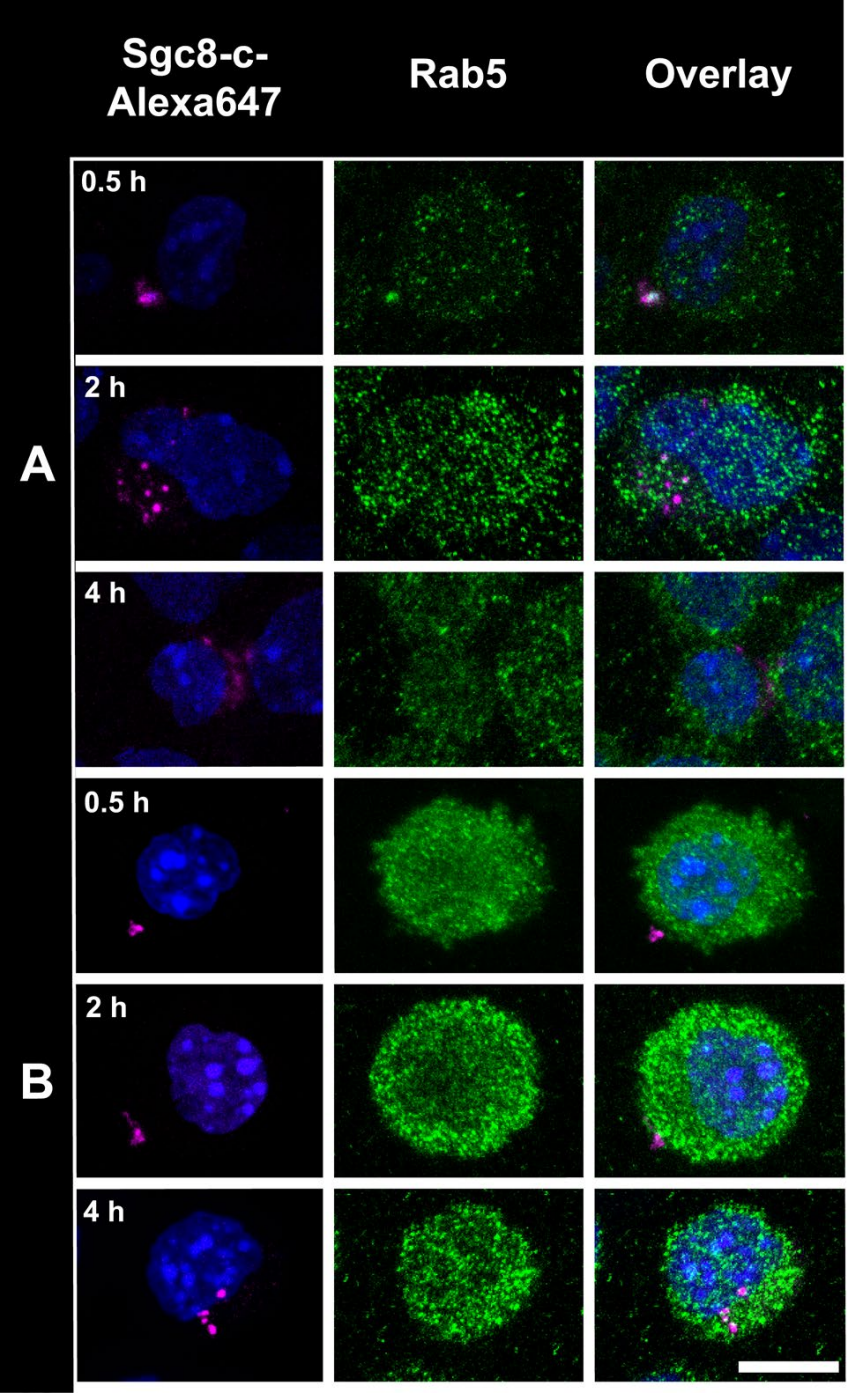
Sgc8-c-Alexa647 co-localize within endosomes. Confocal microscopy of (**A**) B16F10 and (**B**) B16F1 tumor cells incubated for 0.5, 2 and 4 h with the probe. Magenta: Sgc8-c-Alexa647, Blue: *Hoechst* and Green: *Rab5*. Scale bar: 10 µm.

Since it was observed that Sgc8-c-Alexa647 is internalized, we analyzed if the probe was also co-localized within the endosomes like other aptamers-probes^53^. For that, tumor cells were incubated with the fluorescent probe for different times and subsequently the early endosomes were analyzed. Sgc8-c-Alexa647 was observed to co-localize within endosomes in both melanoma cell lines (Fig. 5 and Fig. S2 in Supporting Information). Similarly, it was observed that the signal from the probe showed a tendency towards polarization. This is in agreement with what has been previously seen, that PTK7 is not uniformly distributed in the cell membrane, presenting a dynamic role in cell polarization^49^.

### In vivo biological studies

**Figure 6:**
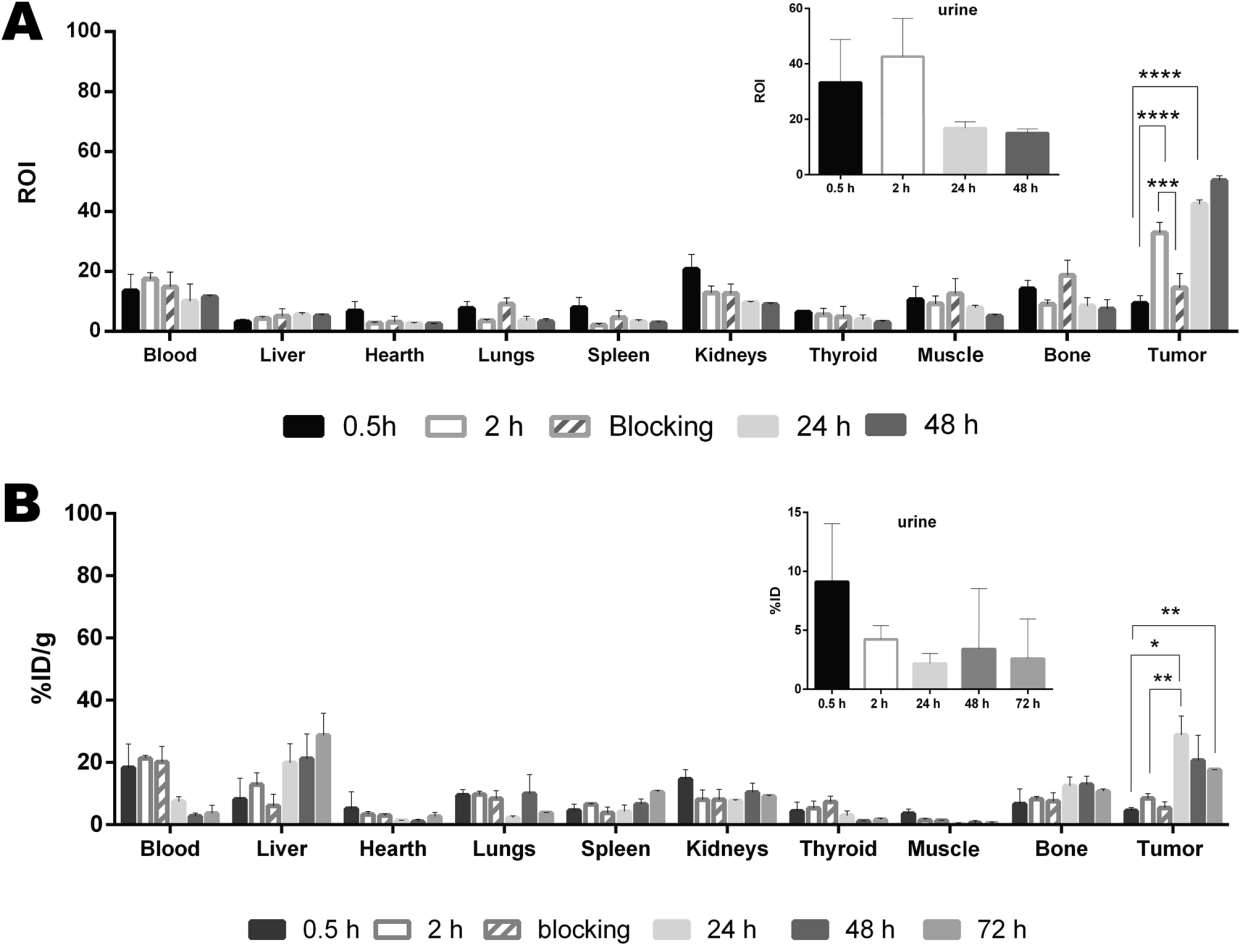
Biodistribution of the probes in B16F10 tumors. (**A**) Biodistribution of Sgc8-cAlexa647 and (**B**) Sgc8-c-NOTA-^67^Ga. ****p < 0.0001; ***p < 0.001, **p < 0.01; *p < 0.05 (Student's *t* test).

**Figure 7:**
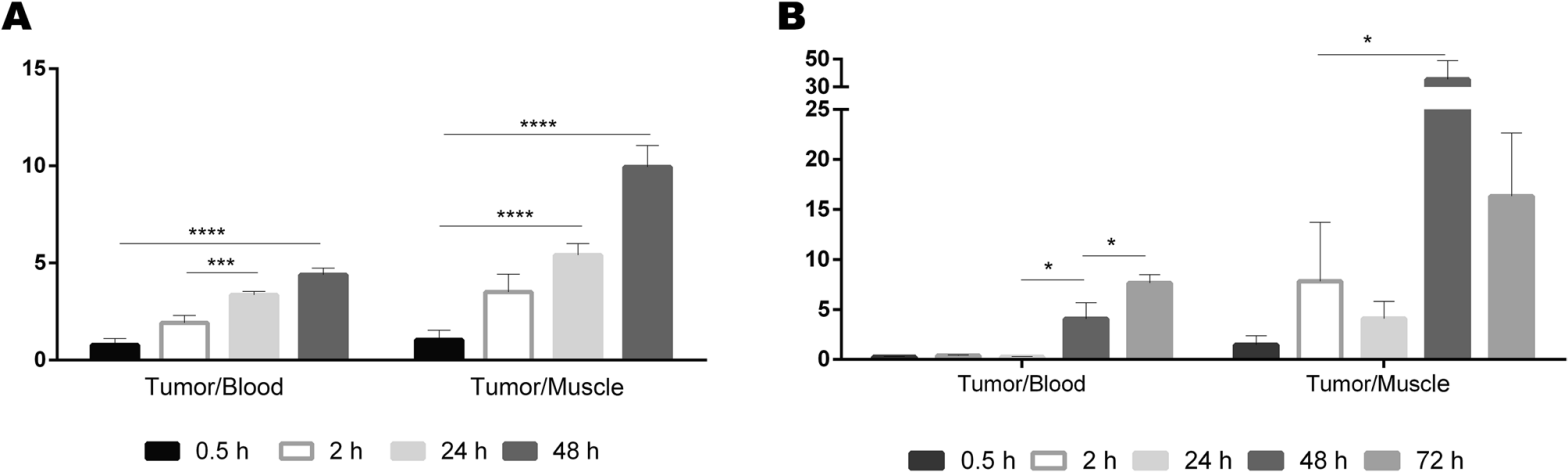
Tumor/blood and tumor/muscle ratios in the tumor model generated with B16F10 cells. (**A**) Sgc8-cAlexa647 and (**B**) Sgc8-c-NOTA-^67^Ga in the tumor model generated with B16F10 cells. ****p < 0.0001, ***p < 0.001, *p < 0.05 (Student's *t* test).

**Figure 8:**
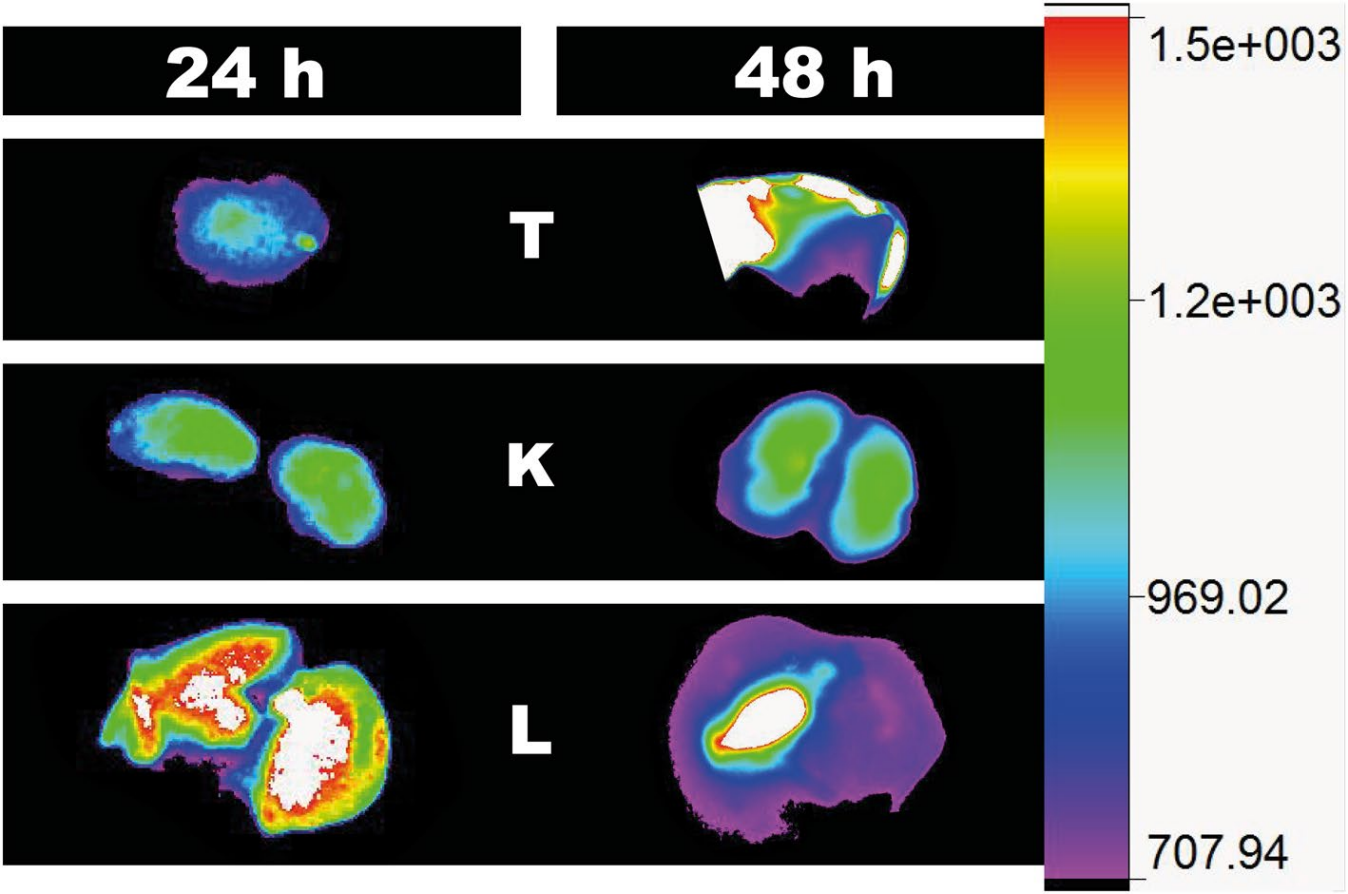
Ex vivo images with Sgc8-c-Alexa647 probe. Tumor (T), kidneys (K) and liver (L) were observed for melanoma tumor model generated with B16F10 cells 24 and 48 h post-injection of the probe.

In vivo studies using both Sgc8-c-Alexa647 and Sgc8-c-NOTA-^67^Ga probes were performed in murine metastatic melanoma model. To do this, once the induced tumors were palpable, probes were i.v. administered, and imaging and biodistribution studies were performed at different times after injection. The results showed interesting characteristics regarding the uptake of the probes in the tumor (Figs. 6, 7). Rapid tissue penetration was visualized, with tumor retention of both probes (Figs. 6, 7). Using the fluorescent probe, a tumor uptake of 32.9 ± 3.5% was observed at 2 h post-injection, increasing significantly at 24 h (42.4 ± 1.5%) and at 48 h (50.3 ± 1.6%) (Figs. 6A, 8). At 2 h post injection of the fluorescent probe, 33.2 ± 15.6% was eliminated in urine, decreasing to 42.6 ± 13.8% at 24 h post-injection (Fig. 6). No significant signal was observed in non-target organs (Fig. 6A). A similar tendence was observed in the non-metastatic melanoma model (Fig. S3).

**Figure 9:**
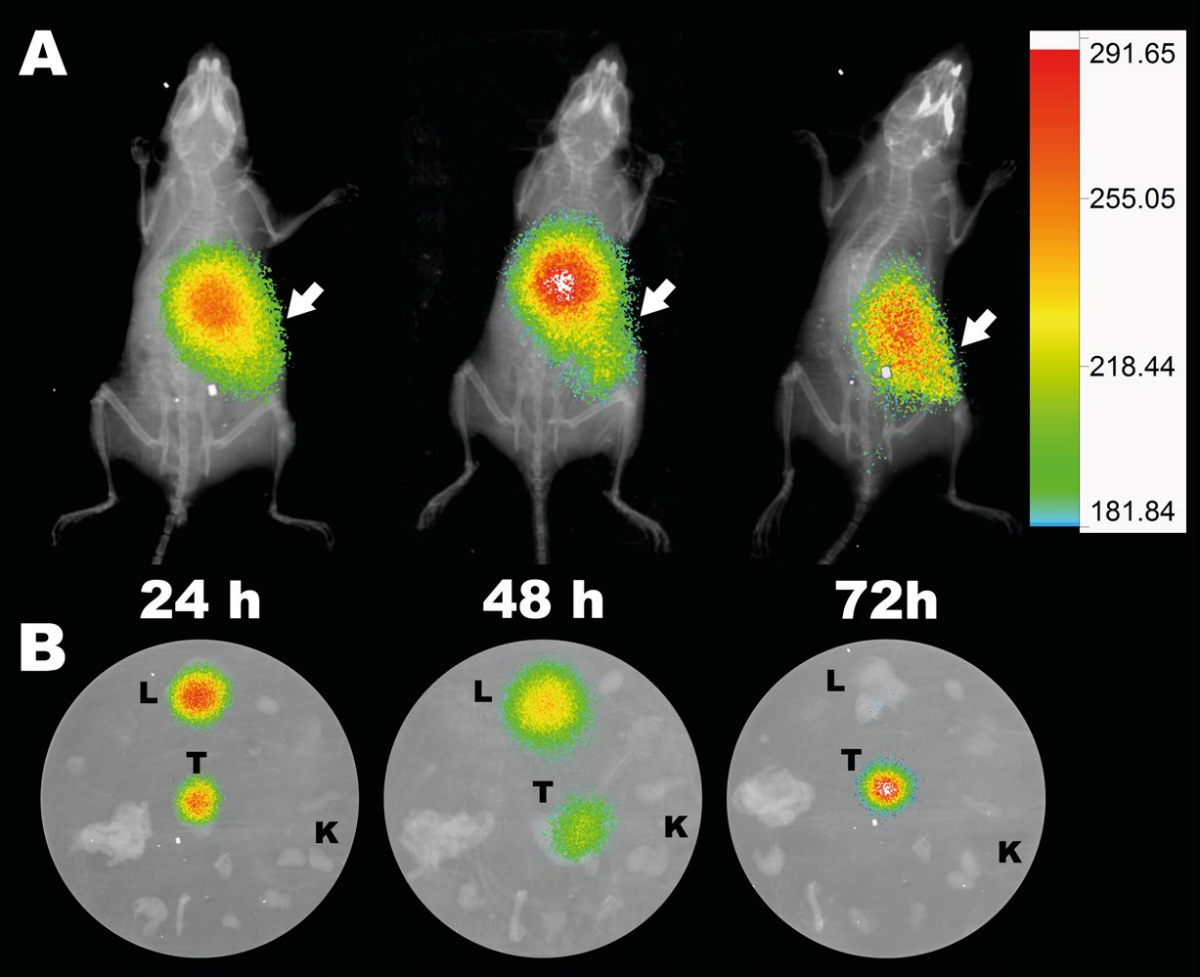
Multimodal images of animals injected with Sgc8-c-NOTA-^67^Ga probe. (**A**) In vivo and (**B**) Ex vivo images at 24, 48 and 72 h post-injection of the probe. Arrow indicates the locations of the tumors. T: tumor, L: liver and K: kidneys. Ex vivo images also include bone, lungs, blood, heart, muscle, intestine, stomach, thyroid, and spleen.

The radiolabeled probe showed an increasing tumor uptake 24 h after injection. At 2 h post-injection, a tumor uptake of 8.4 ± 1.6%ID/g was observed, increasing significantly at 24 h (28.8 ± 6.2%ID/g). Then, at 72 h after injection, tumor uptake decreased slightly (17.7 ± 0.1% ID/g) (Fig. 6B). Urinary excretion was observed at early time points. A value of 9.1 ± 4.9% ID was observed at 0.5 h post-injection, which decreased to 4.2 ± 1.6%ID at 2 h (Fig. 6). These data were determined by the renal values that were due to the elimination of the probes or their metabolites (Figs. 6B, 8). After 24 h post-injection, it was possible to distinguish signals from liver and intestines in the in vivo and ex vivo images, considering hepatobiliary metabolism for later time points (Figs. 6B, 9). However, non-significant signal comes from other organs, which allowed us to observe a clear signal from the tumor (Fig. 9).

Differences in signal values between organs and tumor were evident with both probes, resulting in an optimal tumor/non-tumor organ ratio, having a significant increase over time (Fig. 7). Mainly at 48 h post-injection with radiolabeled probe in the metastatic model, generated by B16F10 cells, showed a tumor/muscle ratio of 35.5 ± 13.7 (Fig. 7B and Fig. S4 for B16F1 model in Supporting Information), which is an excellent value to consider in vivo images. Likewise, it was observed that tumor uptake increased significantly with time in both models (Fig. S5 in Supporting Information). However, it was observed that the tumor uptake was slightly higher in the metastatic melanoma model, generated with the B16F10 cell line, than with the B16F1 non-metastatic melanoma model (Fig. 6 and Fig. S5 in Supporting Information). This difference could be explained by an increase in the expression of PTK7, since it has been seen that in metastatic melanomas, this receptor is one of the tyrosine kinases that participates in the positive regulation of the formation and function of the invadopodia^9^. Also, this difference in the distribution of the probes, which is also observed in the images obtained by confocal microscopy, could be affected by a structural heterogeneity in the tumor vasculature, since it has been seen that the models generated with the B16F10 line show a significant improvement in vascular density^50^.

In vivo blocking studies with Sgc8-c-NH_2_ showed a statistically significant decrease of tumor signal for the fluorescent probe (Fig. 6 and Fig. S3, Supporting information), confirming specific probe training. These assays also reproduce the results seen in hematological models^38^, indicating that there was competition between the probe and the unlabeled aptamer. In addition with the results of the in vivo binding study (Table 1), we confirm the in vivo specific interaction of the probe with PTK7.

## Conclusions

The results described herein together with our previous studies^39,42^, showed that both probes developed with the Sgc8-c aptamer are potential tools for their specific use in the early detection of melanoma. The results of in vitro studies were consistent with those obtained for biodistributions and the in vivo imaging. We obtained interesting characteristics related to the uptake of the probes in the tumor, with optimal tumor/non-target organs ratios, which are global benchmarks that encourage us to continue working on this. Although we found the best ratios at 24 h, other acquisition time points can be evaluated to find a better one. Like antibodies, aptamer-based probes are feasible in the clinic. One of most remarkable results is the low backgrounds versus high uptake in tumor which is very good and convenient. These methodologies applied here allowed us to detect differences in the expression of the tumor marker PTK7 in two different melanoma tumor models. However, it should be studied in depth whether this difference in the expression of PTK7 is really involved in signaling pathways that, consequently, grants greater metastatic power to cells.

The optimal tumor uptakes of the probes in metastatic melanoma, make them promising tools to facilitate in vivo diagnosis and thus to select an appropriate therapy. These probes could also be advantageous for developing intraoperative imaging devices, combined or not, the properties of both probes for use in guided surgeries; identifying and pointing out the tumor margins, helping in the surgical resection of tumors and even helpful in detecting metastases^42,51^. A deepened study with the metastatic melanoma model is currently in progress with the aim to improve the use of probes in early diagnosis that allows the selection of an efficient and personalized therapy, and even for monitoring after remission.

Our results support the potential role of the Sgc8-c-NOTA-^67^Ga and Sgc8-c-Alexa647 as molecular imaging probes optimum to improve strategies in non-invasive molecular diagnosis in melanoma, as well as theranostic approaches.

## Methods

### Synthesis and purification of Sgc8-c-NOTA-^67^Ga and Sgc8-c-Alexa647

The synthesis and purification of both probes were performed following previous reports from our laboratory^39,41,42^.

### In vitro biological studies tumor cell lines

*Mus musculus* melanoma B16F1 (ATCC, CRL-6323), *Mus musculus* metastatic melanoma B16F10 (ATCC, CRL-6475) and *Homo sapiens* glioblastoma U87 MG (ATCC, HTB-14) cell lines were grown in adherence in Dulbecco's Modified Eagle's Medium (DMEM) (Capricorn, Ebsdorfergrund, Germany) supplemented with 10% FBS (Sigma-Aldrich, St. Louis, USA) and 2 mM l-glutamine (Sigma-Aldrich, St Louis, USA). *Homo sapiens*, acute lymphoblastic leukaemia CCRF-CEM (ATCC, CCL-119) cell line was grown in a suspension of RPMI-1640 medium (Sigma-Aldrich, St. Louis, USA) supplemented with 10% FBS and 2 mM l-glutamine. The CCRF-CEM and U87 MG cell lines were used as a positive and negative controls, respectively^52,53^. All cell lines were provided from ATCC (American Type Culture Collection, VA, USA) and were cultured at 37 °C with 5% CO_2_.

### Binding studies for radio-probe

For in vitro cell binding assays, 1.0 × 10^6^ cells from the B16F10 cell line were washed with sterile Phosphate-buffered saline (PBS) pH 7.4, centrifuged at 1000 rpm for 3 min and incubated with 100,000 cpm of the Sgc8-c-NOTA-^67^Ga probe by 0.5, 2 and 4 h at 37 °C. In addition, a competition assay was performed by incubating cells with an excess of underivatized aptamer (Sgc8-c-NH_2_, 5 µg, 0.4 nmol) for 0.5 h a 37 °C. After 0.5 h, these same cells were incubated with the labeled probe for an additional 2 h at 37 °C. At the end of the incubation time, the medium was removed, and cells were resuspended and washed twice with PBS, centrifuging at 1000 rpm for 3 min. The test was done in quintupled. The activity retained in the cells was measured on aGamma counter (PC-RIA MAS, Stratec).

### Binding studies for fluorescent-probe

The murine tumor cell line B16F10, and the human tumor cell lines CCRF-CEM and U87 MG, were washed with sterile PBS pH 7.4, centrifuged at 1000 rpm for 3 min and approximately, 5 × 10^5^ cells were incubated at 37 °C for 30 min with different concentrations of the Sgc8-c-Alexa647 probe (0.007, 0.3 and 1 µM). After incubation, the medium was removed by centrifugation at 1000 rpm for 3 min and washed with PBS, and centrifuging again at 1000 rpm for 3 min. The test was done in quintupled. For each sample, 10,000 events were detected using a 635 nm excitation, laser detector and BP 660/20 nm. The FACS Canto II flow cytometer (BD Biosciences, San Diego, CA, USA) equipment and data were analyzed using FACS Diva and FlowJo software. These results were validated with an anti-PTK7-PE antibody (United States Biological. Clone Type: Polyclonal. Catalog Number: 033359-PE). Specific mean fluorescence indices (MFIs) were calculated as the mean fluorescence of Sgc8-c-Alexa647 cells positive in the presence of aptamer over the mean fluorescence of the entire cell population in the absence of aptamer.

### Western blotting

Supernatants and cell pellets from B16F10 cell cultures were collected separately. Pellets were washed with sterile PBS pH 7.4, centrifuged at 1000 rpm for 3 min and stored at − 20 °C until use. Supernatant proteins were obtained by precipitation with trichloroacetic acid (TCA). Briefly, supernatants were centrifuged for 15 min at 4000*g* at 4 °C recovered and passed through a 0.22 µm filter. Then, TCA (10% final solution) was added and incubated for 1 h on ice. Pellets were obtained by centrifugation, for 30 min at 13,000 rpm and at 4 °C, and further washed three times with 1 mL of acetone and allowed to dry. Finally, proteins were resuspended in 500 µL of PBS pH 7.4. The amount of sample to be used in the Western blot was normalized, quantifying the samples using the bicinchoninic acid assay. Subsequently, these samples were run on a 12% SDS-PAGE gel at 100 V and the blotting membrane was transferred overnight at 400 mA at 4 °C. The membrane was blocked with 5% milk in PBS pH 7.4, for 2 h at room temperature (RT). Subsequently, it was incubated with either the Sgc8-c-Alexa647 probe (10 μg, 0.8 nmol) or the anti-PTK7-PE antibody, at the concentrations recommended by the manufacturer. Three washes with PBS/TBS were performed before the membrane was observed in an imaging equipment (In-Vivo MS FX PRO instrument, Bruker, Billerica, USA).

### Confocal microscopy

To perform confocal microscopy assays, 1 × 10^5^ cells from the B16F10 and B16F1 tumor cell lines were grown on round glass coverslips (12 mm) inside 24-well plates. These cells were incubated with the Sgc8-c-Alexa647 probe (10 μg, 0.8 nmol) for different times (2, 4, and 16 h). Cells were washed with sterile PBS pH 7.4 and fixed with 4% paraformaldehyde. Subsequently, the coverslips were placed in a humid chamber, the cells were blocked with 2% bovine serum albumin in PBS for 20 min at RT and then they were blocked for an additional 15 min, also at RT, with the same solution but adding Triton (0.3%). Cells were incubated for 1 h at RT with the *Hoechst 33342* nuclear marker (1:100, Immuno Chemistry Technologies, LLC) and with the *WGA-green* membrane marker (1:100, thermofisher scientific, USA). They were washed three times with PBS pH 7.4 and three more times with mili Q water. The coverslips were mounted with ProLong^®^ (thermofisher Scientific, USA) and the images were acquired in the confocal microscope LEICATCS-SP5-DMI6000 (HeNe laser, 10 mW: 633 nm). To determine if the probe was internalized endosomically, we followed the same protocol as before, incubating for 0.5, 2 and 4 h and instead of using a membrane marker, the early endosomal marker *Rab5* (rabbit, 1:100, C8B1 mAb 3547, Cell Signaling Technology, USA) was used. The secondary antibody anti-rabbit IgG, Alexa 488-conjugated (goat, 1:500, ab 150077 Abcam, USA) was used.

### In vivo biological studies

#### Animals

Female C57BL/6 mice, 8–12 weeks of age, were used for the in vivo evaluation. Animal experimentation protocols were approved by the Ethical Committee of the University for Animal Experimentation, Uruguay (approval number: 240011-001891-17), all experiments were performed following the principles outlined in the Declaration of Helsinki and complying with the ARRIVE guidelines. Animals were purchased from URBE (Unidad de Reactivos y Biomodelos de Experimentación, Facultad de Medicina-Universidad de la República, Montevideo, Uruguay). Animals were housed in wire mesh cages (racks with filtered air) at 20 ± 2 °C with cycle of 14 h of light and 10 h of darkness. They were fed ad libitum to standard pellet diet and given water ad libitum and were used after a minimum of 3 days acclimation to the housing conditions. Animals were monitored daily, recording their behavior and the presence or absence of tumor. Tumor location and volume was recorded, checking that they did not exceed a diameter of 5 mm. Isoflurane was used for anesthesia and at the end of the experiments the animals were sacrificed by cervical dislocation.

#### Binding studies for fluorescent-probe

For this assay 2.5 × 10^5^ cells/100 mL of the B16F10 cell line were injected subcutaneously into the right flank of C57BL/6 mice. Once tumors were palpable (10–12 days), the Sgc8-c-Alexa647 probe (25 μg, 2 nmol) was injected intravenously (i.v.) through the tail vein. At 0.5, 2 and 24 h post injection, mice were sacrificed to obtain the tumor, liver, spleen and marrow derived from the femur. Organs and tissues were disaggregated by passing through a cell strainer 70 μm (BD Bioscience) and resuspended in sterile PBS pH 7.4. For each sample, 10,000 events were detected using the same laser, detector, and equipment mentioned above. The test was done in quintupled. Data were analyzed using FACS Diva and FlowJo software.

#### Imaging and biodistribution

To generate the melanoma tumor model murine cells B16F1were inoculated in the C57BL/6 mice, as described above for B16F10. Once melanomas were palpable, the Sgc8-c-Alexa647 probe (25 μg, 2 nmol) was injected i.v. and at 0.5, 2, 24 and 48 h post- injection (n = 5 per time group), mice were sacrificed. Ex vivo images of organs (liver, heart, lungs, spleen, kidneys, thyroid, muscle, bone, blood and tumor) were acquired using the imaging equipment above mentioned, with the X-ray and fluorescence model. The results were expressed in ROI and the tumor/blood and tumor/muscle ratios were calculated. In vivo specificity was evaluated in a competition test. One group of mice (n = 5) was first i.v. injected with Sgc8-c-NH_2_ in excess of 5 times more than the probe. After 30 min post-injection, the same mice were i.v. injected with the Sgc8-c-Alexa647 probe. After 2 h mice were sacrificed, images acquired and organs weighed. Similar evaluation was performed for the radiolabelled probe. Sgc8-c-NOTA-^67^Ga was i.v. injected (~ 1850 kBq) and biodistribution of the probe was followed until 72 h post-injection. Over time, the animals were anesthetized with isoflurane to perform the images in vivo. Live images were acquired for 0.5, 2, 24, 48 and 72 h after injection by X-rays andGamma modalities in the imaging equipment. For the biodistributions animals were sacrificed, images were acquired and the levels of radioactivity in the tissue were measured using a Gamma counter (PC-RIA MAS, Stratec). Radioactivity levels were expressed as percentage of injected dose per organ gram (%ID/g) and injected dose (%ID). Competitive blocking assays were also performed, injecting mice first with 0.5 nmol of Sgc8-c-NH_2_ and after 30 min re-injected with Sgc8-c-NOTA-^67^Ga and at 2 h the imaging and biodistribution studies were performed.

#### Statistical analysis

Statistical analysis was performed using the Student's *t* test because the data are independent and have a normal distribution. The *p* values of significance are indicated in each figure.

## Supplementary Information

Supplementary Figures.
