# Supplementary material for: Metastatic and non-metastatic melanoma imaging using Sgc8-c aptamer PTK7-recognizer
Source: Sci Rep. 2021 Oct 7;11:19942. doi: 10.1038/s41598-021-98828-6 (PMC8497559; doi:10.1038/s41598-021-98828-6)
Supplement: Supplementary file 1 — Supplementary Figures. [file 41598_2021_98828_MOESM1_ESM.doc]

**SUPPLEMENTARY MATERIAL**

Metastatic and non-metastatic melanoma imaging using Sgc8-c aptamer PTK7-recognizer

Estefanía Sicco^1,2^, Amy Mónaco^2^, Marcelo Fernandez^3^, María Moreno^2^, Victoria Calzada^1,*^, and Hugo Cerecetto^1^

^1^Área de Radiofarmacia, Centro de Investigaciones Nucleares, Facultad de Ciencias, Universidad de la República, 11400 Montevideo, Uruguay
^2^Departamento de Desarrollo Biotecnológico, Instituto de Higiene, Facultad de Medicina, Universidad de la República, 11600 Montevideo, Uruguay
^3^Laboratorio de Experimentación Animal, Centro de Investigaciones Nucleares, Facultad de Ciencias, Universidad de la Republica, 11400 Montevideo, Uruguay
^*^vcalzada@cin.edu.uy

| **Figure S1 ………………………………………………………………………..…..** | **S1** |
| --- | --- |
| **Figure S2 ………………………………………………………………………..…..** | **S2** |
| **Figure S3 ………………………………………………………………………..…..** | **S3** |
| **Figure S4 ………………………………………………………………………..…..** | **S4** |
| **Figure S5 ………………………………………………………………………..…..** | **S5** |
|  |  |


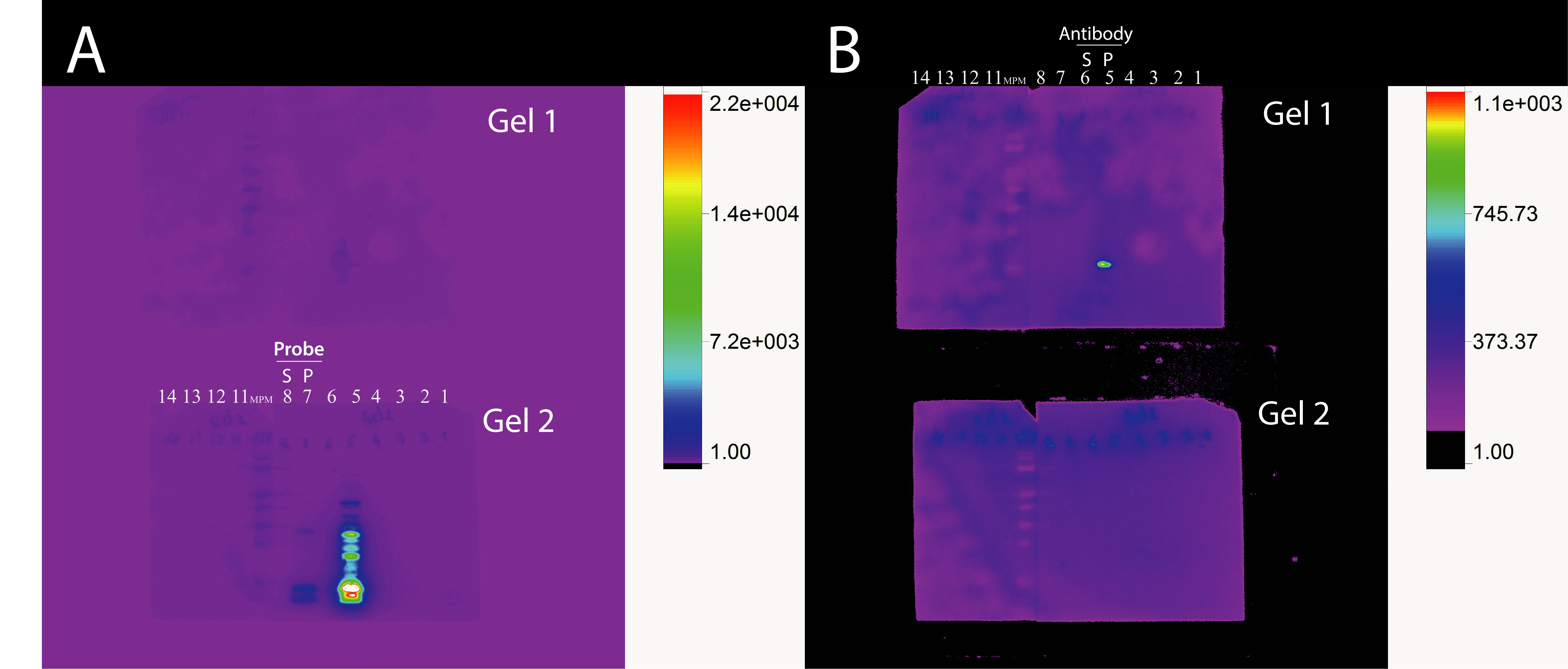


**Figure S1. Full-length gels of proteins extracted from the supernatant (S) and pellet (P) of B16F10 cells culture.** Exposition was performed with **A.** the Sgc8-c-Alexa647 probe, or **B.** the anti-PTK7-PE antibody. Gel 1 has been incubated with the antibody and Gel 2 with the probe. Both gels show the grouping of Western blot cropped of proteins extracted from the supernatant (S) and pellet (P) of B16F10 cells culture, used to generate Figure 1B of the article. Gel 1 lanes (from left to right): 14-11, negative control (samples incubated without the probe); 9, molecular weight marker; 8, B16F10 cell supernatants; 7, B16F10 cell pellets; 6-3, A20 cell supernatants and pellets. Gel 2 lanes (left to right): 14-11, negative controls (samples incubated without the antibody); 9, molecular weight marker; 6, B16F10 cell supernatants; 5, B16F10 cell pellets.

**
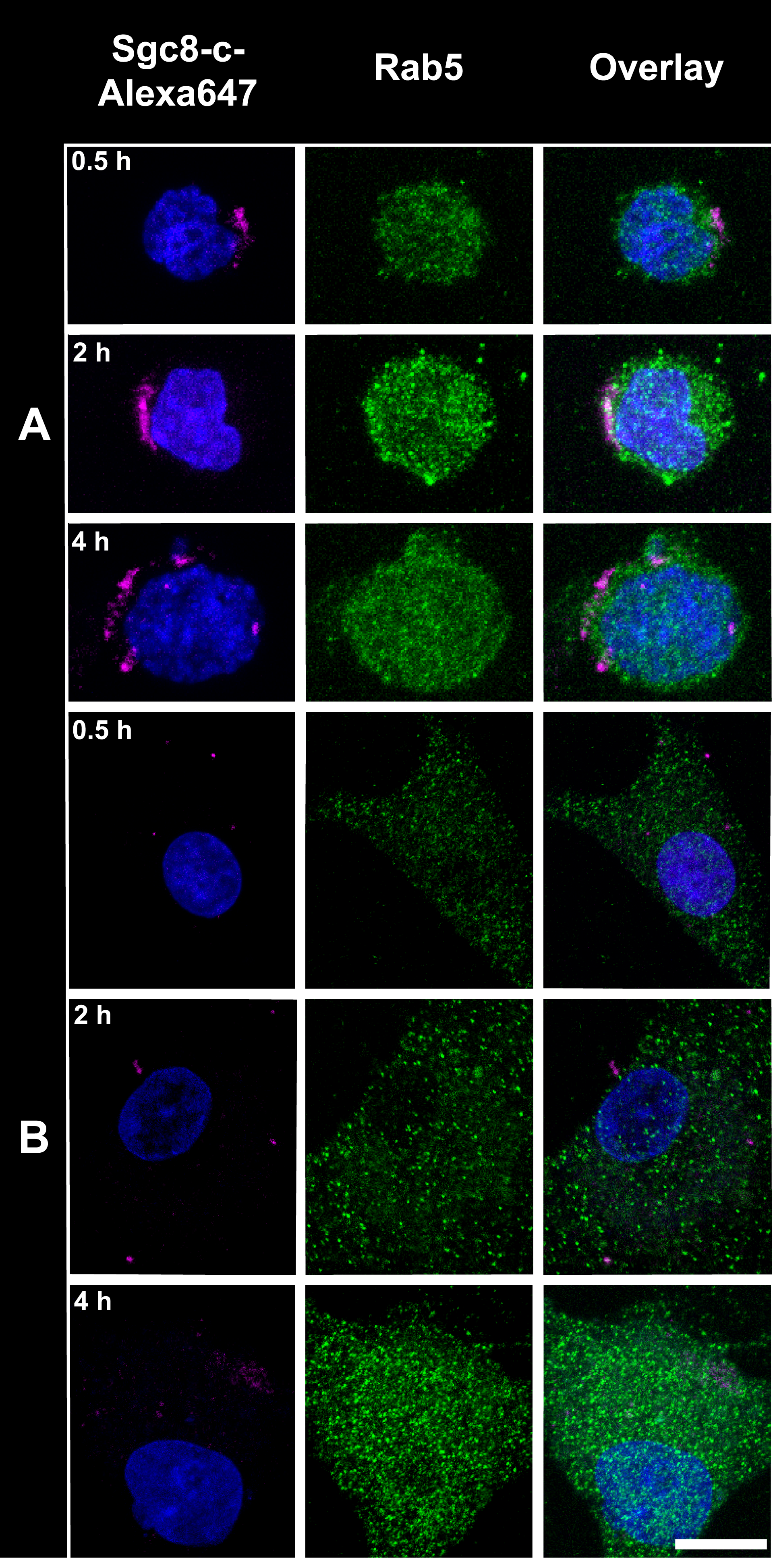
**

**Figure S2. Sgc8-c-Alexa647 co-localize within endosomes.** Confocal microscopy of **A.** CCRF-CEM and **B.** U87MG tumor cells incubated for 0.5, 2 and 4 h with the probe. Magenta: Sgc8-c-Alexa647, Blue: *Hoechst* and Green: *Rab5.* Scale bar: 10 µm.

**
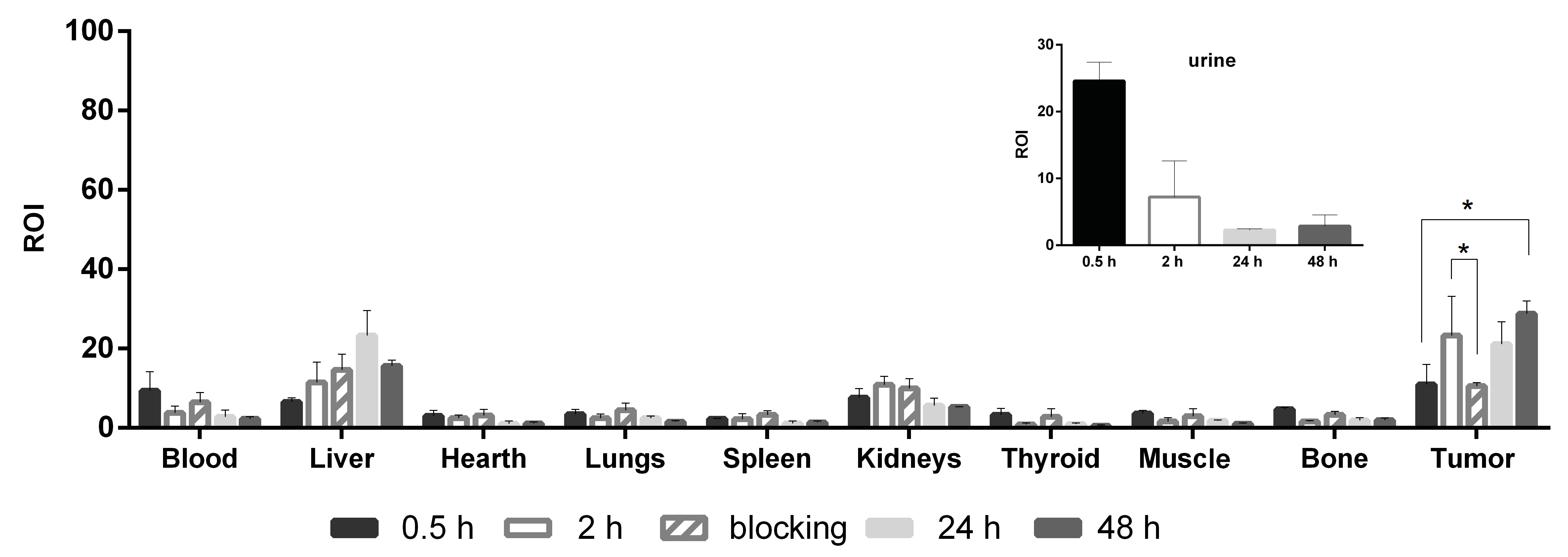
**

**Figure S3. Biodistribution of Sgc8-c-Alexa647 probe in B16F1 tumors.** * p < 0.05 (Student's t-test).


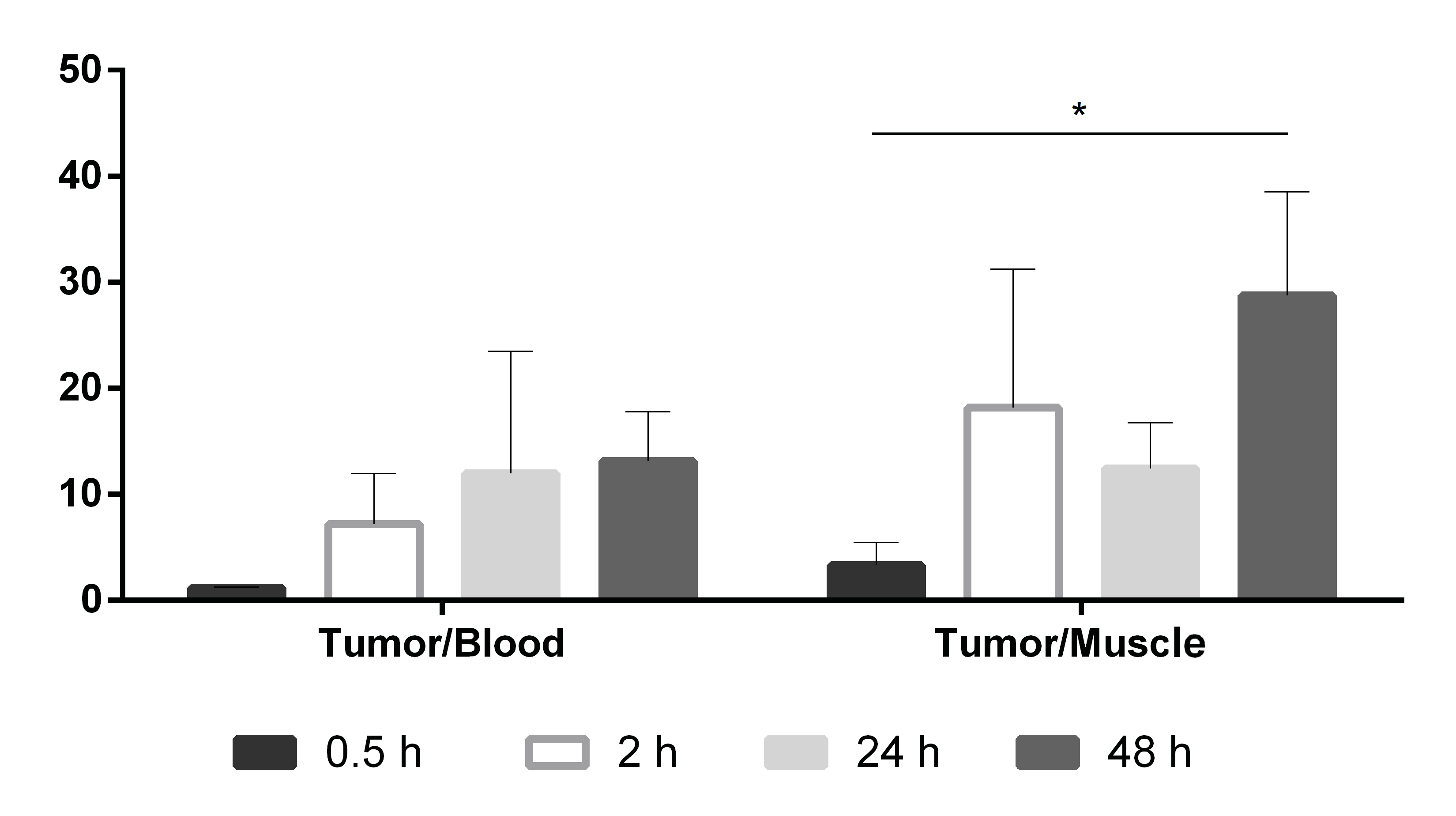


**Figure S4. Tumor/blood and tumor/muscle ratios in the tumor model generated with B16F1 cells using Sgc8-cAlexa647 probe.** * p < 0.05 (Student's t-test).


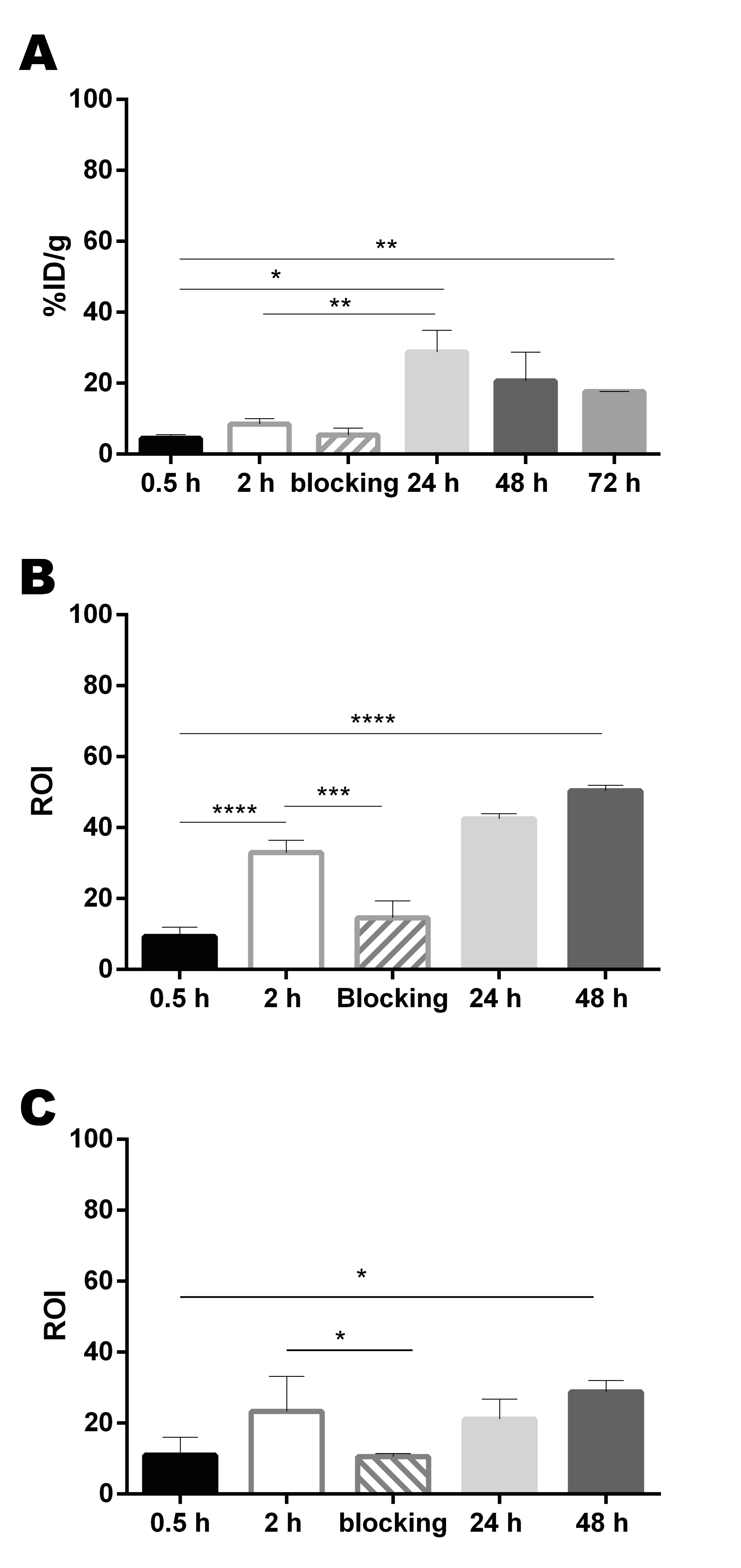


**Figure S5. Biodistribution of the probes in tumor.** In **A.** Biodistribution of Sgc8-c-NOTA-^67^Ga and in **B.** Sgc8-cAlexa647 in the tumor model generated with B16F10 cells. In **C.** Biodistribution of Sgc8-cAlexa647 in the tumor model generated with B16F1 cells. **** p < 0.0001, *** p < 0.001, ** p < 0.01, * p < 0.05 (Student's t-test).
